# Theoretical lateral and axial sensitivity limits and choices of molecular reporters for Cherenkov-excited luminescence in tissue during x-ray beam scanning

**DOI:** 10.1117/1.JBO.25.11.116004

**Published:** 2020-11-12

**Authors:** Ethan P. M. LaRochelle, Brian W. Pogue

**Affiliations:** Thayer School of Engineering, Dartmouth College, Hanover, New Hampshire, United States

**Keywords:** Cherenkov-excited luminescence, Cherenkov emissions, tissue optics, Monte Carlo modeling, radiation therapy, medical physics, cloud computing

## Abstract

**Purpose:** Unlike fluorescence imaging utilizing an external excitation source, Cherenkov emissions and Cherenkov-excited luminescence occur within a medium when irradiated with high-energy x-rays. Methods to improve the understanding of the lateral spread and axial depth distribution of these emissions are needed as an initial step to improve the overall system resolution.

**Methods:** Monte Carlo simulations were developed to investigate the lateral spread of thin sheets of high-energy sources and compared to experimental measurements of similar sources in water. Additional simulations of a multilayer skin model were used to investigate the limits of detection using both 6- and 18-MV x-ray sources with fluorescence excitation for inclusion depths up to 1 cm.

**Results:** Simulations comparing the lateral spread of high-energy sources show approximately 100× higher optical yield from electrons than photons, although electrons showed a larger penumbra in both the simulations and experimental measurements. Cherenkov excitation has a roughly inverse wavelength squared dependence in intensity but is largely redshifted in excitation through any distance of tissue. The calculated emission spectra in tissue were convolved with a database of luminescent compounds to produce a computational ranking of potential Cherenkov-excited luminescence molecular contrast agents.

**Conclusions:** Models of thin x-ray and electron sources were compared with experimental measurements, showing similar trends in energy and source type. Surface detection of Cherenkov-excited luminescence appears to be limited by the mean free path of the luminescence emission, where for the given simulation only 2% of the inclusion emissions reached the surface from a depth of 7 mm in a multilayer tissue model.

## 1 Introduction

**Fig. 1:**
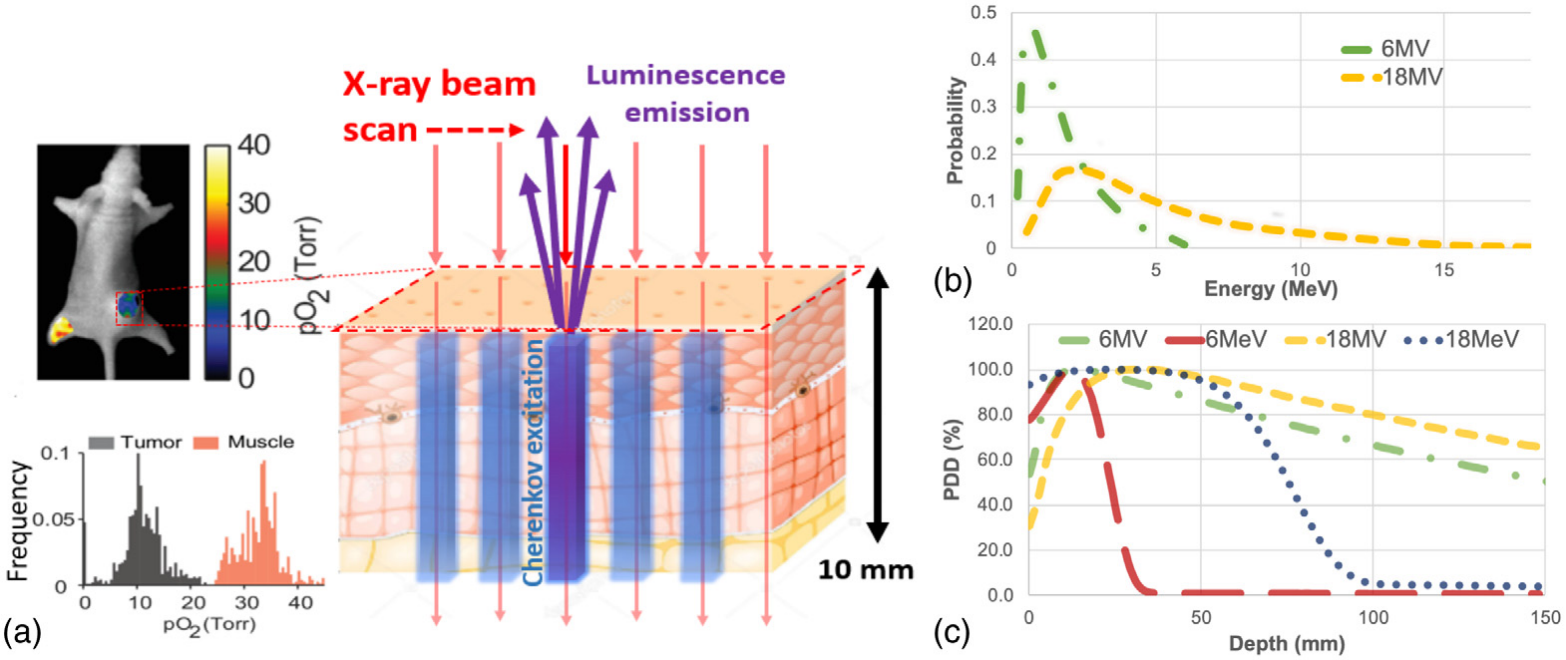
Schematic illustration (a) of an *in vivo* application of Cherenkov-excited luminescence where Cherenkov light is generated at depths into tissue. In this illustration, a mouse with a hypoxic flank tumor and normal muscle tissue are injected with an oxygen-sensitive phosphorescent compound. As the x-ray beam passes through the tissue, Cherenkov emissions occur and excite the luminescent compound. *In vivo* imaging can resolve the depth-integrated voxels, and the resulting estimates can be tabulated into a histogram to describe the heterogeneous extracellular oxygen concentration.^5^ The photon energy distribution used in subsequent Monte Carlo simulations is shown in (b) which determine the characteristics of the Cherenkov intensity and depth within the tissue. The percentage depth-dose (PDD) curves for 10  cm×10  cm photon (6 and 18 MV) and electron (6 and 18 MeV) beams in water are shown in (c) where electrons have a higher chance of interaction and deposit dose more superficially, whereas photon beams must first generate a high-energy electron through Compton scattering before Cherenkov emissions can occur. Cherenkov emissions are correlated with dose, so the PDD can be used as an estimate of the depth distribution of the optical emissions.

Cherenkov-excited luminescence has previously been demonstrated as a method to improve the depth sensitivity of *in vivo* optical imaging^1^^–^^4^ and could be an alternative to optical imaging with fluorescence in deeper penetrance. An example application of Cherenkov-excited luminescence is to excite an oxygen-sensitive luminescent compound during radiation therapy utilizing only the radiation as an excitation source and a sensitive camera for detection, as shown in Fig. 1(a). In conventional *in vivo* fluorescence imaging, utilizing an excitation laser or LED light, there is an exponential decay of the source as it propagates into tissue dictated approximately by the effective attenuation coefficient, $\mu_{\mathrm{eff}}$, defined by diffusion theory as $\mu_{\mathrm{eff}}=\sqrt{3\mu_{a}(\mu_{a}+\mu_{s}^{\prime })}$ where the latter coefficients are for absorption and transport reduced scattering, respectively.^6^ In Cherenkov excitation, the excitation light is produced throughout the volume directly proportional to the dose of the radiation beam for electrons above the 220 keV threshold, following the same build up and fall off with depth, as shown in Fig. 1(c). While in both cases, laser or Cherenkov excitation, the light still has to escape the tissue and is therefore attenuated exponentially by $\mu_{\mathrm{eff}}$ on the way out at the emission wavelength bands, there is still a major benefit from having the exciting light within the volume of tissue. Yet, in comparing optical excitation to radiation beam excitation, it is hard to clearly quantify the benefits for Cherenkov, and so in this study, the (i) spatial resolution, (ii) depth sensitivity, and (iii) optimal fluorophores for Cherenkov excitation, are each examined computationally with Monte Carlo simulations.

One method to improve spatial resolution of Cherenkov-excited luminescence images is to utilize known information about the beam geometry, which has previously been accomplished experimentally by delivering thin sheets of x-rays.^1^^,^^2^ This method applies a deconvolution kernel to account for the beam shape, assuming an XY Gaussian distribution of the Cherenkov emissions corresponding to the multileaf collimator (MLC) leaf opening,^1^ and also accounting for the depth dependence of the Cherenkov emissions.^2^ Both the lateral resolution (XY-spread) and axial sensitivity (or depth, Z-dependence) of Cherenkov emissions rely on a number of factors, predominantly the beam shape and energy of the beam, and the optical properties of the object being imaged. In addition, since Cherenkov emissions are correlated with dose, but not a direct measurement of dose, deconvolution can also be applied to improve the spatial resolution of the estimated delivered dose based on images of surface Cherenkov emissions.^1^^,^^7^^,^^8^

In this work, Monte Carlo modeling of the Cherenkov emission and Cherenkov-excited luminescence were carried out with a GEANT4 GAMOS plugin that included optical interactions.^9^^,^^10^ To make the simulations as realistic as possible for human or animal studies, a seven-layer skin model^11^^,^^12^ was used to estimate Cherenkov emission spread resulting from the delivery of a narrow x-ray or electron beams. Expanding on this approach, additional adipose tissue and muscle were added to the model geometry to compare the limits of detection for wide-field optical illumination and Cherenkov-excited luminescence.

Finally, the unique convolution of the Cherenkov spectrum, $I_{\mathrm{Ch}}(l)\approx 1/\mu^{2}$ where μ represents wavelength, with the tissue optical interactions with $\mu_{a}(\mu )$ and $\mu_{s}/(\mu )$, results in a complex broad spectrum light source, peaked more in the red than the blue-green wavelengths. The light could still be used to excite a number of fluorophores *in vivo*, and so to better understand the optimal utility, the resultant *in situ* spectrum was modeled in conjunction with a chemical database to define which Cherenkov-excited luminescent compounds might be ideal based upon the tumor composition. The overall goal of this work was to demonstrate how Monte Carlo simulations can help define the limits of sensitivity within the complex interactions occurring during this form of imaging and then inform improved deconvolution kernels or choice of molecular probes.

## 2 Materials and Methods

Two sets of simulations were performed using the GAMOS 6.1 tissue optics plugin.^9^^,^^10^ This Monte Carlo modeling package combines high-energy physics engines for handling the ionizing radiation interactions, with a tissue optics plugin that provides the ability to track optical emissions and propagation. The two simulation sets run in this modeling software investigated the lateral spread of Cherenkov emissions from thin sheet sources of photons and electrons and also investigated the depth of sensitivity for detecting a luminescent inclusion excited by either Cherenkov emissions or optical epi-illumination.

### 2.1 Cherenkov Lateral Resolution

A skin equivalent geometry based on a seven-layer skin model^11^^,^^12^ was used to estimate the spread of Cherenkov emission. This model simulated a 2.5  mm×10  mm thin sheet of high-energy photos or electrons incident on the skin geometry and originated 0.2 m above the tissue volume. The minimum width of 2.5 mm chosen as this is the minimum MLC thickness available in commercial clinical linear accelerators (LINAC) and so provides the minimum beam lateral thickness available without a custom setup. A total of $10^{7}$ events were generated for each photon energy of 6 and 18 MV, as well as electron energies of 6 and 18 MeV, with these values being chosen based upon them being the minimum and maximum energies available on most clinical LINACs. The energy distribution of the photon beams is shown in Fig. 1(b). The photon simulations were split into 10 separate simulations, and the electron simulations were split into 100. Each simulation was given a unique random seed and executed using a cloud infrastructure.^10^ Each photon simulation required ∼30 to 50 min to execute depending on the energy level, whereas each electron simulation required 15 to 30 min. The initial and final position of each optical Cherenkov emission was recorded.

Experimental measurements were collected with an intensified CMOS camera (C-Dose Research, DoseOptics LLC, Lebanon, NH) where a 3  mm×40  mm sheet of 6 and 18 MV photons was delivered into water in a clear plastic container with the water surface at isocenter. Using the 6×6 electron beam collimator with a custom 3  mm×40  mm Cerrobend insert as a collimator, electron energies of 6, 9, 12, and 18 MeV were also imaged. The camera was positioned such that it was level with the surface of the water. A 500-nm short-pass filter (500FL07-50, Andover Corp, Andover NH) was attached to the front of a 50-mm f/1.2 lens. The camera was place on the couch ∼0.7  m from the water container. Images were processed using Python 3.7 and scikit-image 0.16.2.

### 2.2 Limits of Depth Sensitivity and Detection

**Fig. 2:**
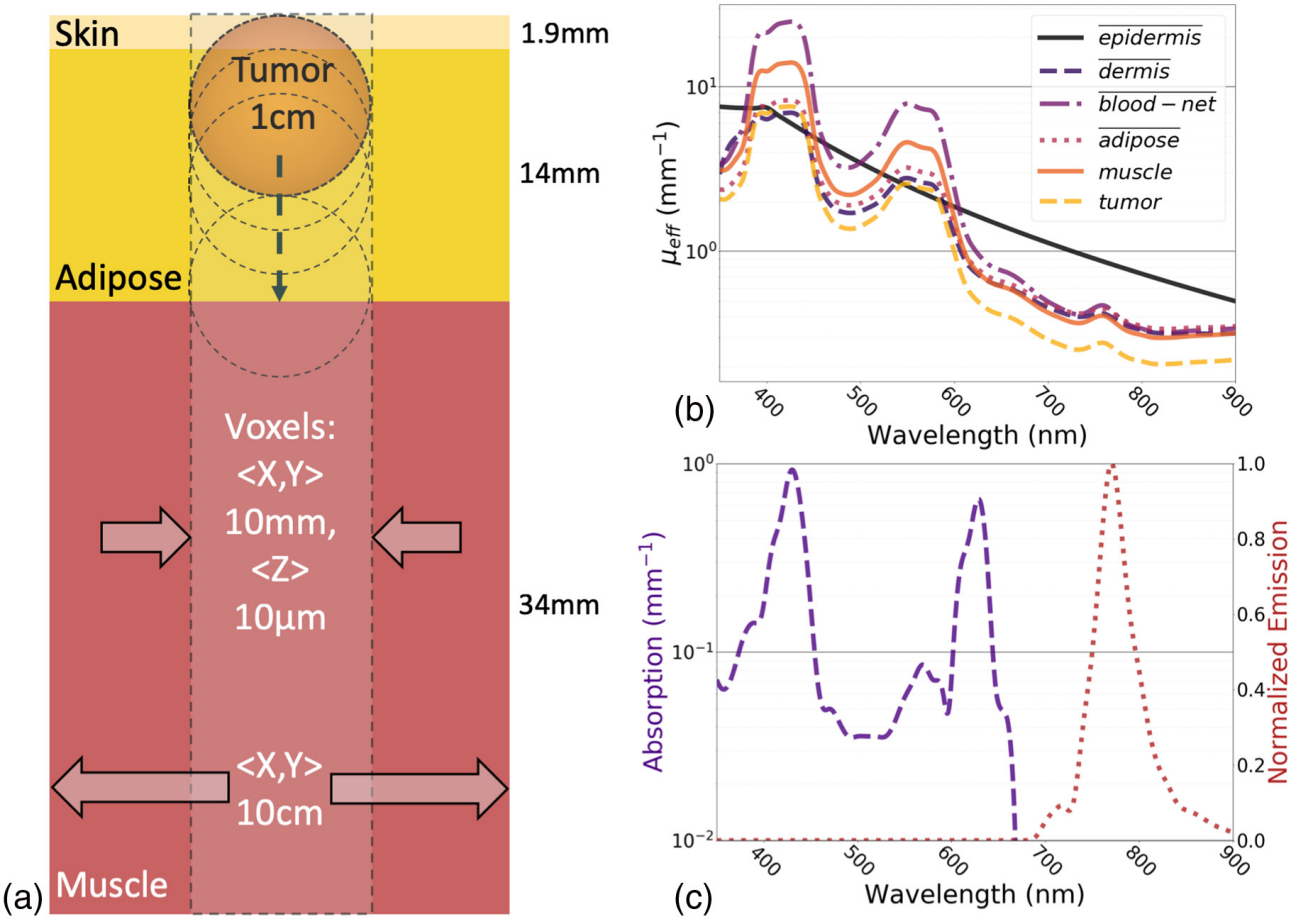
A multilayer tissue model containing (a) a tumor-simulating inclusion is defined where (b) the average $\mu_{\mathrm{eff}}$ is shown for each layer-type. (c) The luminescent absorption and emission assigned to the tumor is provided as a contrast agent.

A model with tissue-equivalent geometry containing an inclusion with fluorescence contrast was defined, where the inclusion depth and primary excitation source were varied using input arguments. The multilayer tissue-equivalent geometry based on a seven-layer skin model^11^^,^^12^ was combined with additional layers of adipose and muscle.^13^^–^^15^ The surface dimensions for each layer were 10  cm×10  cm, and the entire tissue geometry was 5 cm in depth (Fig. 2). The properties of the seven-layer skin model have been documented in previous publications^11^^,^^12^ and are summarized in Fig. 2(b). Each layer has a specific density and mixture of predefined materials as well as optical properties $\left(\mu_{a},\mu_{s},g,n\right)$ for wavelengths between 350 and 900 nm. A 1-cm diameter spherical inclusion was also defined with a set of optical properties and additional fluorescence absorption and emission characteristics, which most closely resemble the oxygen-sensitive luminescent compound PtG4,^16^ which has been used in many previous experimental studies.^1^^,^^4^ This molecule has absorption peaks near 430 and 630 nm with luminescence emission near 770 nm, with a lifetime that is effected by the local oxygen concentration in tissue.

Simulations of a 1-cm square source with 0.97 deg divergence placed 0.9 m above the tissue geometry were run with $10^{7}$ optical photons (430 and 630 nm) and the same number of x-ray photons (6 and 18 MV). With the beam divergence, the total area of the incident events on the tissue surface is ∼4  cm×4  cm. Tumor inclusion depths measured from the top of the tumor to the tissue surface were defined between 0 and 10 mm. A parallel world voxel geometry was defined with 1  cm×1  cm×10  μm voxels used to measure light fluence and for high-energy sources, dose deposited. Additional filters and detectors were defined to determine the position and wavelength of Cherenkov and fluorescence emissions starting in the tumor inclusion, exiting the tumor inclusion, and reaching the surface. Simulations for the 6- and 18-MV source were split into 10 simulations of $10^{6}$ events for each tumor inclusion depth, whereas the lower complexity of the 430- and 630-nm optical excitation simulations allowed for single $10^{7}$ event simulations to be executed in a short amount of time. Experimental comparison of the luminescent tumor geometry was not performed, but *in vivo* applications have been considered previously.^3^^–^^5^

The spectral characteristics of the Cherenkov emissions in the tumor inclusion (Fig. 2) were compared to the absorption spectrum of compounds in the PhotoChemCAD database.^17^^,^^18^ This database is publicly available and contains the absorption and emission spectra and quantum yield of many compounds. The entire database can be downloaded in the form of a text file. This file contains a list of chemicals and their properties and links to files containing the absorption, and emission spectra, as well as links to images of the compound’s chemical structure. A Python script was written to parse this text file and the corresponding absorptions files. All compounds with reported absorbance between 350 and 850 nm were considered for analysis. The Cherenkov spectra in the tissue, $I_{\mathrm{Ch},\text{tissue}}(l_{i})$, was interpolated and resampled to match the reported absorption spectra of each compound $j,m_{a,j}(l_{i})$. The product of these two spectra was integrated between the previously stated bounds and the result was multiplied by the quantum yield, $\varphi_{QY}$, as described here

$$S_{j}=\underset{i}{\sum }I_{\mathrm{ch},\text{tissue}}(l_{i})*m_{a,j}(l_{i})*\varphi_{QY}.$$

The result was a scalar ranking of signal, $S_{j}$, where all values were normalized to the highest ranked compound.

## 3 Results

### 3.1 Cherenkov Lateral Resolution

**Fig. 3:**
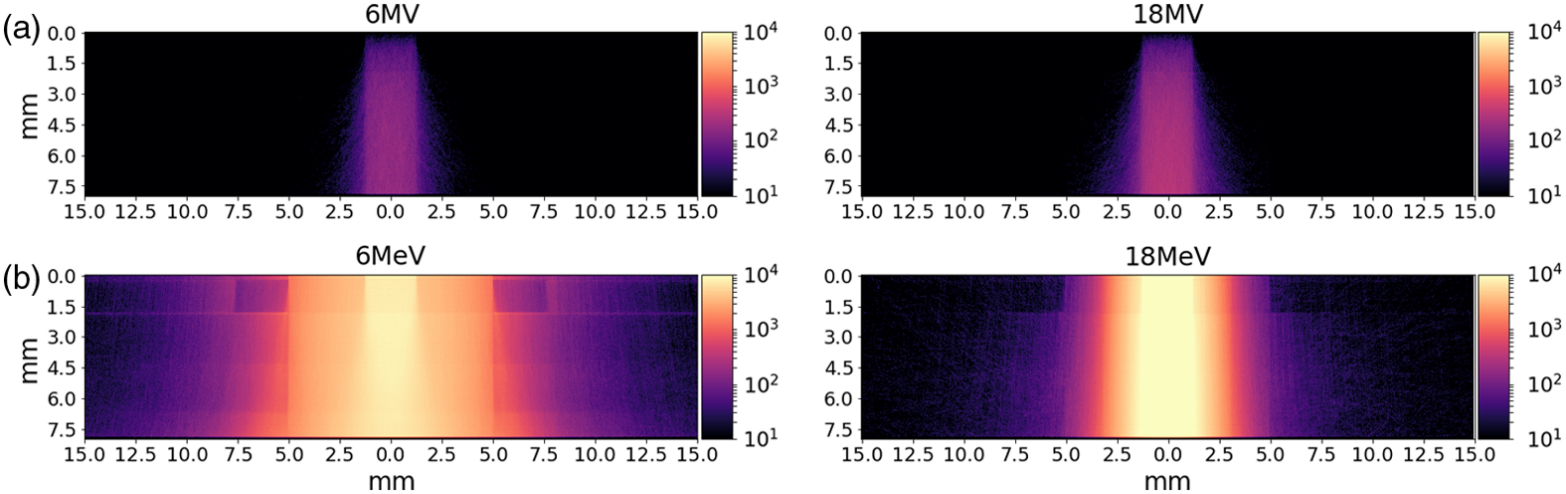
Cherenkov emissions recorded from simulations of (a) 2.5-mm wide photon source or (b) electron source in multilayer tissue volume.

Text files recording the initial and final position of Cherenkov emissions were generated using filter detectors in the GAMOS simulations. While the simulation was a 3D geometry, the XZ positions were tabulated, effectively reducing the dimensionality of the data. The resulting photon counts generated from $10^{7}$ initial events are shown in Fig. 3. From these simulations, it can be observed there are ∼100× more Cherenkov emissions generated due to the electron source. This is expected due to the higher energy of the electrons compared to the photon energy distribution and because each electron at this high energy level will result in numerous Cherenkov emissions, whereas high energy photons must first undergo a scattering event before electrons are freed. These simulations also indicate the electron source would have a wider penumbra. In the simulations, this effect is more pronounced in lower energy electron sources, mainly due to the shorter mean free path and higher likelihood of electron scattering, as has been shown.^19^^,^^20^ The lower optical emissions in the first 1.5-mm of tissue are likely due to the higher absorption coefficients, whereas below this depth the volume is defined as adipose tissue.

**Fig. 4:**
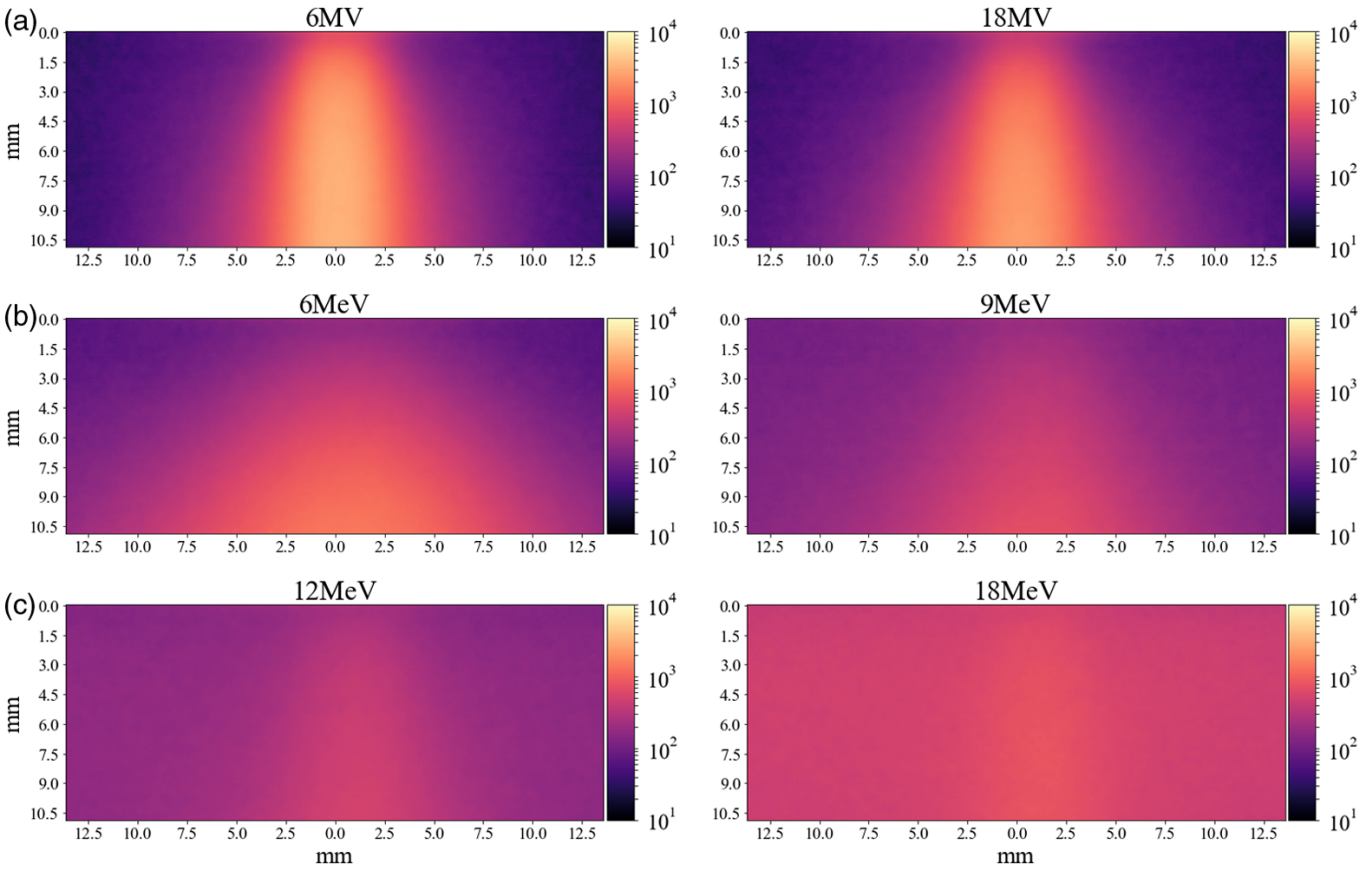
Experimental Cherenkov measurements of (a) photon and (b) and (c) electron beams in water.

Cherenkov imaging of water was performed as a comparison to the simulated results. Images were collected with both photons and electrons and results are shown in Fig. 4. From these images, it can be observed the photon source has largely similar distributions within the first 10 mm of water. However, the Cherenkov emission distributions of the electron sources are highly energy dependent.

**Fig. 5:**
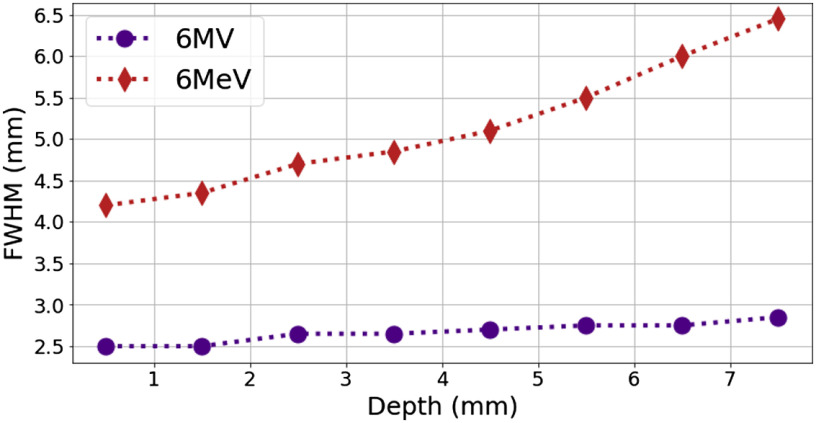
Analysis of beam width as a function of depth for 2.5-mm wide photon (6 MV) and electron (6 MeV) sources. The average FWHM was calculated for each millimeter of depth in the tissue.

The simulation data and experimental images were analyzed to determine the full width at half maximum (FWHM) for each for each millimeter of depth. The experimental and simulation data cannot be directly compared but show similar tends of larger FWHM for electron sources. The analysis for the simulations is shown in Fig. 5, where the electron sources have a wider FWHM as a function of depth, likely due to the increased probability of interaction. Even at depths beyond 5 mm there is minimal beam divergence observed for the photon source.

### 3.2 Limits of Depth Sensitivity and Detection

**Fig. 6:**
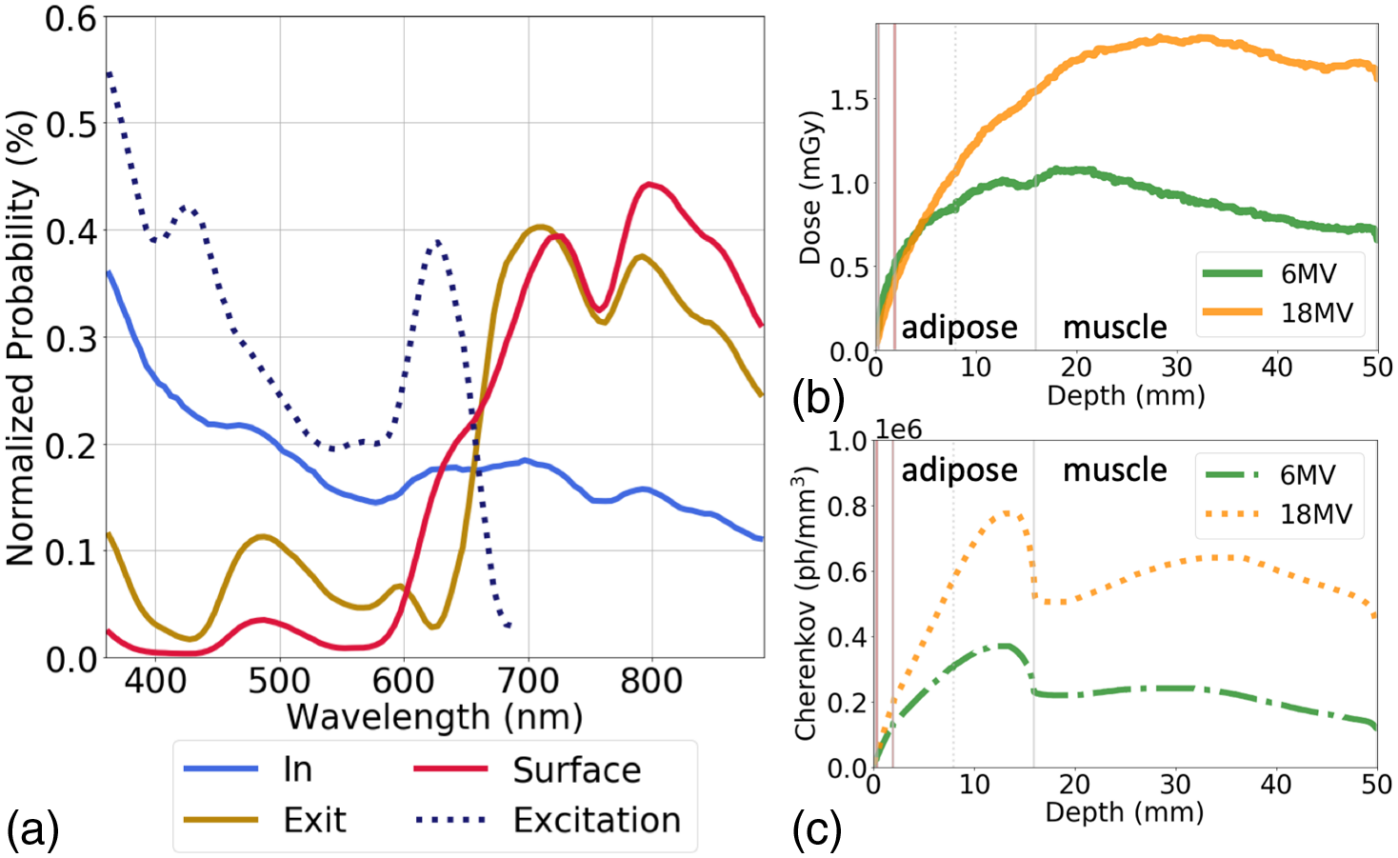
(a) The spectral distribution of optical emissions in a model with a tumor inclusion at depth of 2 mm, as detected in the tumor (light blue), exiting the tumor (orange), at the surface (red), or contributing to luminescent excitation (dotted indigo). (b) The deposited dose for 6 and 18 MV at depths into the tissue model and (c) the corresponding optical Cherenkov fluence, which is clearly affected by the tissue optical properties of each layer.

Using detector filters to monitor the track of Cherenkov emissions, the spectral distribution can be monitored at various locations in the model. The spectral emissions are expected to follow a $1/\lambda^{2}$ distribution,^21^ which is generally observed in the tumor, with small increases at areas of lower attenuation, likely due to emissions originating outside the tumor and then being counted when entering the tumor [Fig. 6(a)]. As these emissions exit the tumor, another detector tabulated the spectral characteristics, showing a small peak around 480 nm while the majority is distributed beyond 600 nm, due to the longer mean free path in this spectral region. A similar long-pass filtering effect is seen at the surface, where the emissions are mainly in the red-NIR range. While the tumor fluorescence absorption is modeled after PtG4 [Fig. 2(c)], the $1/\lambda^{2}$ spectral distribution of the Cherenkov emissions must also be considered. This model provides the ability to determine the actual excitation wavelength distribution of the Cherenkov-excited luminescence, as shown in Fig. 6(a) (dotted-line).

The Cherenkov emissions are related to the deposited dose which increases with depth into the tissue, where the maximum dose ($D_{\text{max}}$) is expected to be 1.5 cm for 6 MV and 3.5 cm for 18 MV photon beams which is similar to our simulation results [Fig. 6(b)]. While in a homogeneous medium, Cherenkov emissions are thought to be directly correlated with deposited dose, Fig. 6(c) shows how the higher attenuation of muscle and blood can greatly decrease the overall light fluence, which corresponds with previous studies of Cherenkov emissions in the presence of tissue optical properties.^22^^–^^24^

**Fig. 7:**
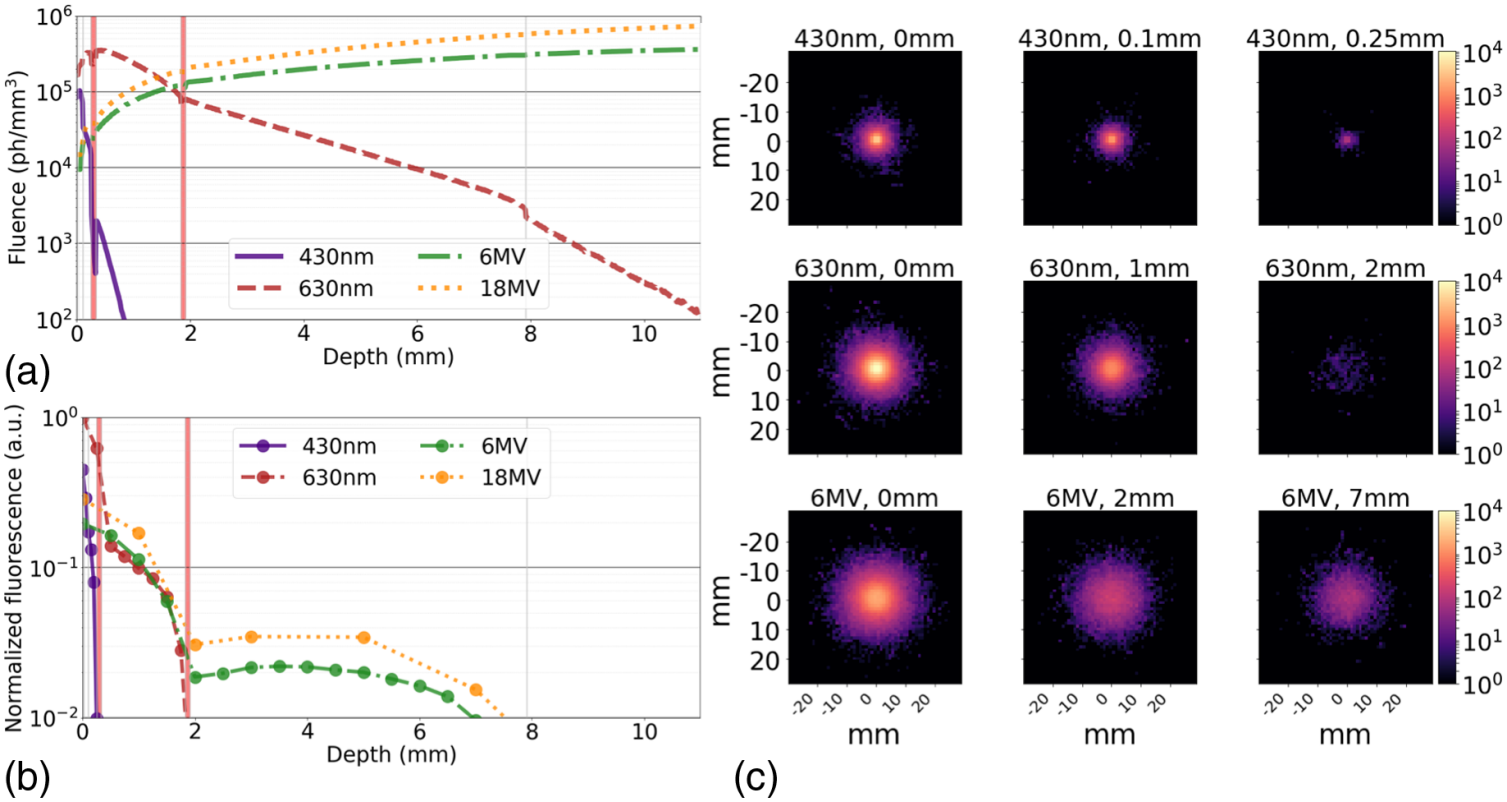
(a) The photon fluence due to external optical illumination or x-ray induce Cherenkov emissions at depth in tissue is compared to the corresponding normalized fluorescence detected at the surface, where (b) the blood networks (red vertical lines) introduce considerable attenuation. (c) 2D-histograms of the surface luminescence.

As expected, these simulations of multilayer a tissue volume show the fluence rate for the 430-nm source drops significantly in under 1 mm, and the 630-nm source follows suit after a few millimeters [Fig. 7(a)]. The light gray vertical lines in Fig. 7(a) show the skin layer boundaries, with light red-shaded regions indicating blood networks in the skin, which correspond with sharp drops in fluence due to the high absorption of these layers. By placing a detector at the surface, all fluorescence emissions exiting the tissue volume could be counted. GAMOS has the ability to count the origination of all fluorescent photons and those exiting the tumor, which shows ∼90% of the fluorescent emissions leave the tumor, but those reaching the tissue surface are much lower (0% to 23%) and dependent on tumor depth. In our model, at a tumor depth of 7 mm, just over 2% of fluorescent photons reach the surface for both 6 and 18 MV. A comparison of surface fluorescence emission intensity for excitation by either 430 nm, 630 nm optical, or a 6-MV x-ray source is provided in Figs. 7(b) and 7(c). A plot of the mean photon count at the surface, relative to the peak value observed by 630 nm excitation, is plotted by tumor depth [Fig. 7(b)], where the depth indicates the distance between the surface and the top of the 1-cm diameter inclusion. Here, it can be observed the 430-nm source has the poorest depth sensitivity of ∼250  μm, while the 630-nm excitation source has improved depth sensitivity of ∼2  mm, and in these simulations, the Cherenkov depth sensitivity is ∼7.5  mm. The sharp drop in depth sensitivity appears to be limited by the absorption of the blood networks, which is ∼2× higher in absorption at the fluorescence peak (770 nm) compared to the surrounding tissue. While the Cherenkov excitation shows the greatest depth sensitivity, the 430-nm excitation source provides the highest potential lateral resolution due to its short path length and resulting smaller tumor cross section [Fig. 7(c)].

### 3.3 Choice of Optimal Fluorescent Agents

**Fig. 8:**
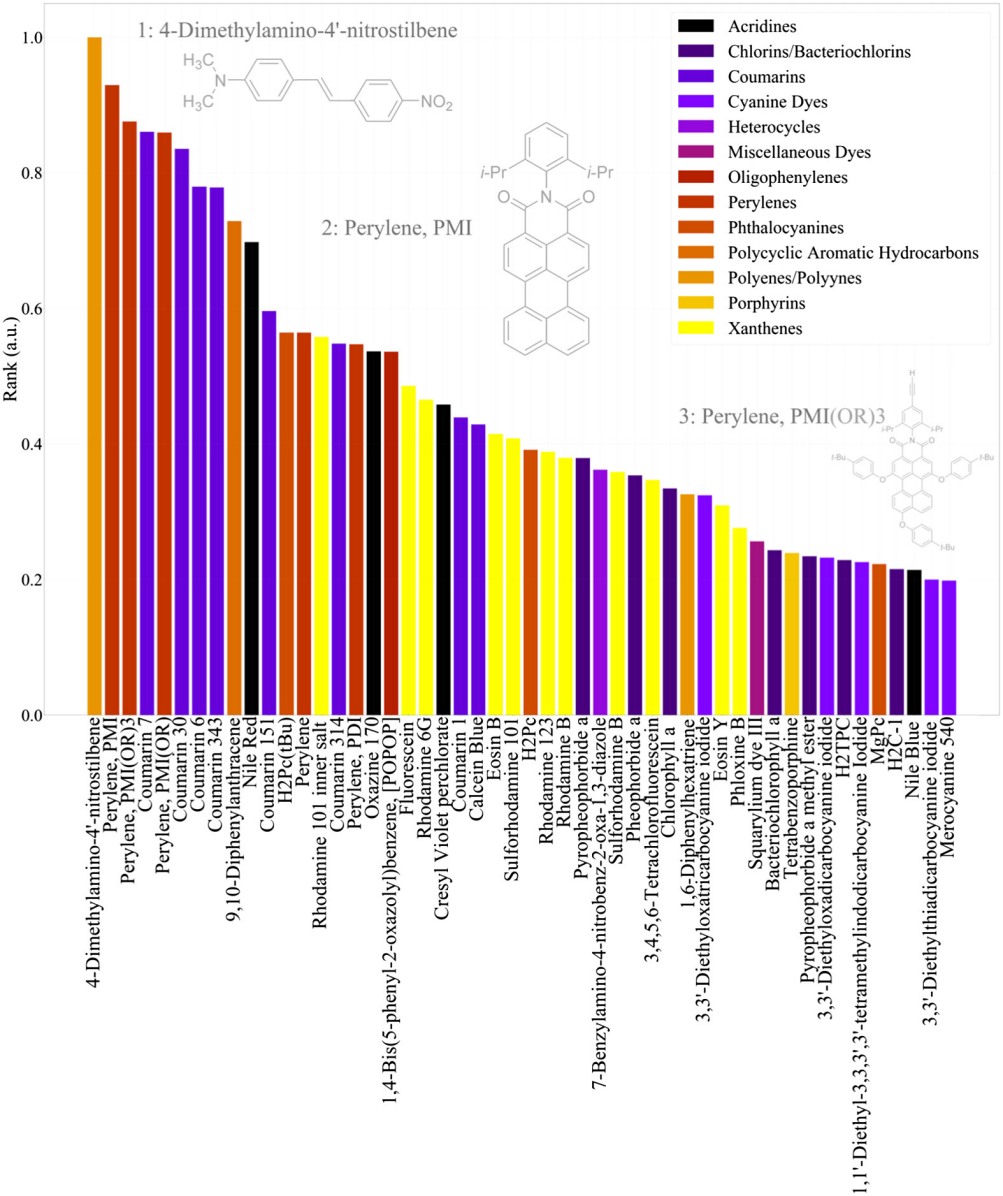
The Cherenkov-excitation ranking of the top 50 candidate compounds available in the PhotoChemCAD database. The chemical structure of the top three candidates is shown.

To identify other potential fluorophores that would be ideal candidates for Cherenkov excitation, a computational ranking of fluorescent compounds was performed. These rankings, the product of the *in vivo* Cherenkov spectra, fluorescence absorption spectra, and quantum yield of each candidate compound are shown in Fig. 8.

**Fig. 9:**
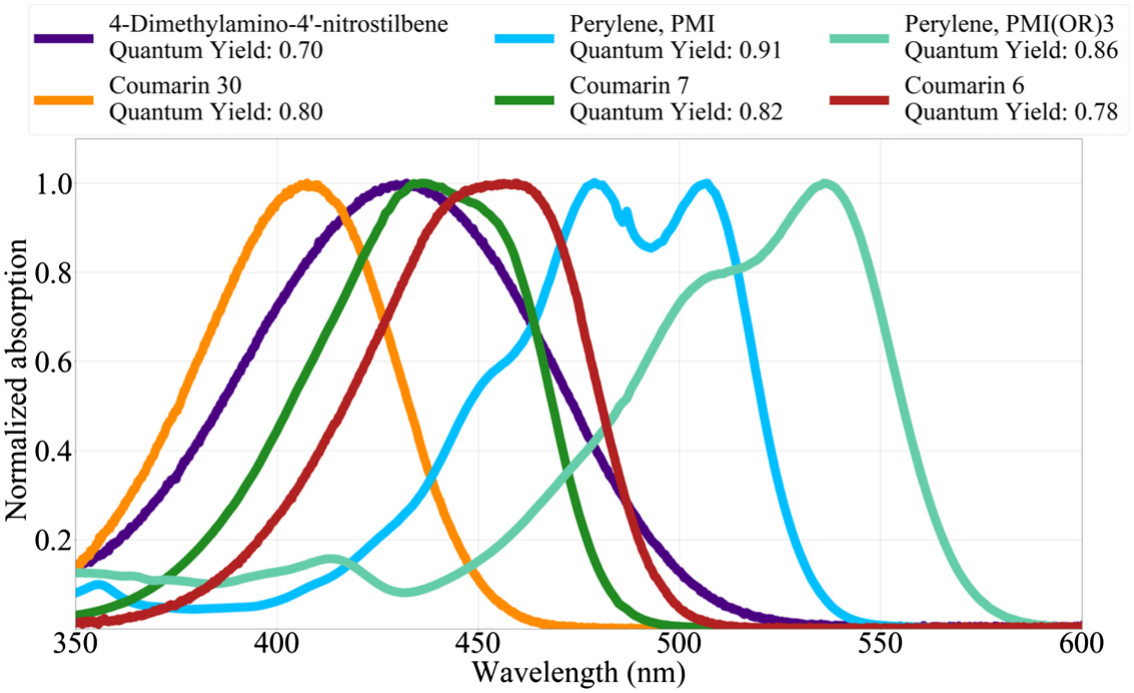
The absorption spectra of compounds with the highest Cherenkov-excitation ranking.

The PhotoChemCAD database organizes each compound into a chemical class, which are color-coded in the above figure, which presents the top 50 candidates. An alternative method of using a $1/\lambda^{2}$ spectral distribution for the Cherenkov emissions produces similar results where the ranking for a few of the compounds is rearranged by one to two places. These rankings do not take into account biocompatibility or lifetime which are generally on the order of nanoseconds for these compounds. The system does not consider the emissions spectra or how it would interact with tissue optical properties or detector quantum efficiency, although these could be implemented using the given data. The absorption spectrum of the top-ranked compounds is shown in Fig. 9.

## 4 Discussion

### 4.1 Cherenkov Lateral Resolution

Electron sources are generally used to provide surface dose due to the increased probability of electron scatter resulting in short penetration depths. Since electrons are charged particles, as they travel through tissue they can exhibit three types of interactive forces: soft collisions, hard collisions, or Coulomb–force interactions. Soft collisions are the most common and occur when the incident electron passes at a considerable distance to an atom and can result in Cherenkov emissions.^25^ In this case, the electron loses very little energy and continues in an undisturbed trajectory. With a hard collision, the incident electron interacts directly with an atom’s electron which is often ejected, often generating a characteristic x-ray.^25^ In Coulomb–force interactions, the incident electron interacts primarily with an atom’s nucleus and is elastically scattered; however, in a small percentage of cases, an inelastic interaction occurs and the electron transfers most energy to the atom, resulting in an x-ray photon emission, also known as a bremsstrahlung emission.^25^

The variety of photon and electron interactions with matter, which often cascade, results in a spreading of the Cherenkov emissions. Developing a model of the spread function is important to improve spatial localization for the delivered dose or for Cherenkov-excited scanned images. While this work demonstrates how simulations can be developed for developing these models, there are improvements that need to be made to the simulation before a deconvolution kernel with clinical utility can be defined. A very similar investigation has been previously conducted by Brost et al. that determined the Cherenkov scatter function in a multilayer tissue model for a number of LINAC energies.^8^^,^^26^

In general, for scanning images utilizing shaped beams, photon sources are required due to the delivery mechanisms of LINACs. If shaped beams are not required, either photon or electron sources could be used to for wide-field excitation.

### 4.2 Limits of Depth Sensitivity and Detection

A series of simulations were developed to better understand the limits of detection for a luminescent inclusion at varying depths in tissue. These simulations compared the light fluence from an external optical source as well as Cherenkov-emissions generated within the multilayer tissue model. While the purely optical simulation for measuring fluence could be performed in a package such as Monte Carlo multilayer,^27^ the physics-engine required for Cherenkov emissions as well as the ability to simulate fluorescence is missing.

While Cherenkov emissions are correlated with deposited dose, these simulations show how tissue optical properties will influence the observed emissions. It is difficult to measure the spectral properties of Cherenkov emission inside tissue, and so these simulations provide insight on how tissue acts as a long-pass wavelength filter, allowing mainly red and NIR to pass to the surface for detection, even though the signal generated inside the tissue is broadband. While an 18-MV beam generates ∼2.5× more Cherenkov emissions than the 6-MV counterpart, since the maximum dose occurs at 3.5 cm much of these optical photons are absorbed before reaching the tissue surface, which is in agreement with previous studies.^23^^,^^28^

Cherenkov-excited luminescence was also compared to purely optical luminescence in these example simulations. It has been reported previously red or NIR light from Cherenkov emissions are actually the predominant wavelength available for luminescence excitation.^23^ While our simulation was able to demonstrate this, it also shows that the UV-blue contribution is as important for the given compound’s excitation [Fig. 6(a)]. While it is well known 630-nm light will have a much higher penetration depth than 430 nm excitation, the complexity of Cherenkov emissions within tissue complicates depth estimates. In the present example, the 6-MV and 630-nm sources had largely similar luminescence reaching the surface when the tumor inclusion was 1 mm below the surface, but when the inclusion depth was moved to 5 mm, the Cherenkov excitation resulted in a surface luminescence over 10× that of the 630-nm excitation. Models such as the one provided in this work could be help provide insight on how multimodal luminescence imaging could be used for improved depth discrimination.

While electron sources are not commonly used for Cherenkov-excited luminescence imaging, their depth-dose profile aligns well with the optical mean free path in tissue, where the energy could be used to adjust the depth of Cherenkov emissions. Since Cherenkov photons are generated along the path of high energy electrons as they pass through tissue, localizing the origin of excited emissions with high depth-resolution is challenging. A combination of multimodal optical and high-energy electron or photon excitation could be used to increase resolution along the z axis. If imaging is not the primary application of Cherenkov excitation, and instead applications such as x-ray or Cherenkov-excited photodynamic activation are desired, then high energy photons would provide the highest depth penetrance.

A system for computational ranking potential compounds to be used in Cherenkov-excited luminescence imaging was demonstrated. While similar datasets are available for scintillators,^29^ neither data sources consider long-lived phosphoresce (>10  μs). If the current available data were expanded and standardized to report lifetime and scintillation yield, the currently demonstrated method could easily be expanded to identify additional means of contrast, such as those described in our previous work.^30^ In addition, if this functionality could be expanded to include triplet quantum yields in addition to fluorescence quantum yields, this computational search could be expanded to identify potential Cherenkov-excited or x-ray-excited photosensitizers. While many of the compounds identified by this work would not be appropriate for the time-delayed phosphorescent imaging as those used for oxygen estimations,^4^^,^^5^ the basis demonstrates a method to quickly identify potential compounds based on empirical data. From the given data, a number of coumarins appear to be suitable for detection by Cherenkov-excitation. When the quantum efficiency of the photocathode is considered,^31^ a blue-sensitive camera is ideally suited to detect emissions from compounds such as Auramine O, coumarins, and curcumin, whereas with a red-sensitive photocathode Nile Blue, Perylenes, and Crystal violet are more appropriate.

## 5 Conclusions

The simulation environment utilized in this work allows for a number of system variables to be tested simultaneously, or for simulations to be easily split into smaller chucks, allowing for faster execution. Using this system, models of Cherenkov spread in tissue and limits of detection for Cherenkov-excited luminescence were demonstrated. Models of thin x-ray and electron sources were compared with experimental measurements, showing similar trends in energy and source type. The limits of detection of a luminescent inclusion were also demonstrated through modeling. From these models, it was demonstrated 630 nm excitation and Cherenkov excitation have similar surface remittance from an inclusion at 1 mm depth, but when the depth is increased to 5 mm, the Cherenkov-excited luminescence at the surface is 10× higher than that generated by 630 nm excitation. In addition, at an inclusion depth of 7 mm, ∼2% of the luminescence generated in the tumor reaches the surface. These models provided further evidence of the filtering effects introduced by tissue and could be used as a basis for improving spatial accuracy and depth sensitivity of Cherenkov imaging systems. A method for computationally ranking potential Cherenkov-excited compounds was demonstrated as a means for quickly identifying potential contrast agents in future experimental work.
